# A roadmap to human hippocampal neurogenesis in adulthood, aging and AD

**DOI:** 10.21203/rs.3.rs-4469965/v1

**Published:** 2024-05-30

**Authors:** Orly Lazarov, Ahmed Disouky, Mark Sanborn, Mostafa Mostafa, K. Sabitha, Aimee Schantz, Namhee Kim, Szymon Pawlowski, William Honer, David Bennett, Yi Zhou, C. Keene, Mark Maienschein-Cline, Jalees Rehman

**Affiliations:** The University of Illinois at Chicago; The University of Illinois at Chicago; University of Illinois at Chicago; The University of Illinois at Chicago; The University of Illinois at Chicago; University of Washington; Rush University; The University of Illinois at Chicago; The University of British Columbia; Rush University Medical Center; Institute of Neuroscience, Chinese Academy of Sciences; University of Washington; University of Illinois at Chicago; University of Illinois at Chicago

## Abstract

In the rodent, hippocampal neurogenesis plays critical roles in learning and memory^1,2^, is tightly regulated by inhibitory neurons^3–7^ and contributes to memory dysfunction in Alzheimer’s disease (AD) mouse models^8–10^. In contrast, the mechanisms regulating neurogenesis in the adult human hippocampus, the dynamic shifts in the transcriptomic and epigenomic profiles in aging and AD and putative niche interactions within the cellular environment, remain largely unknown. Using single nuclei multi-omics of postmortem human hippocampi we map the molecular mechanisms of hippocampal neurogenesis across aging, cognitive decline, and AD neuropathology. Transcriptomic and epigenetic profiling of neural stem cells (NSCs), neuroblasts and immature neurons suggests that the earliest shift in the characteristics of neurogenesis takes place in NSCs in aging. Cognitive impairment was associated with changes in neuroblast profile. In AD, there was a widespread cessation of the transcription machinery in immature neurons, with robust downregulation of genes regulating ribosomal and mitochondrial function. Further, there was substantial loss of parvalbumin+ inhibitory neurons in the hippocampus in aging. The number of the rest of inhibitory neurons were reduced as a function of age and diagnosis. Notably, a similar system-level effect was observed between immature and inhibitory neurons in the transition from aging to AD, manifested by common molecular pathways that were ultimately lost in AD. The numbers of neuroblasts, immature and GABAergic neurons inversely correlated with extent of neuropathology. Using CellChat and NeuronChat, we inferred the ligands and receptors by which neurogenic cells communicate with their cellular environment. Loss of synaptic adhesion molecules and neurotransmitters, either sent or received by neurogenic cells, was observed in AD. Together, this study delineates the molecular mechanisms and dynamics of human neurogenesis, functional association with inhibitory neurons and a mechanism of hippocampal hyperexcitability in AD.

## Introduction

**Figure 1:**
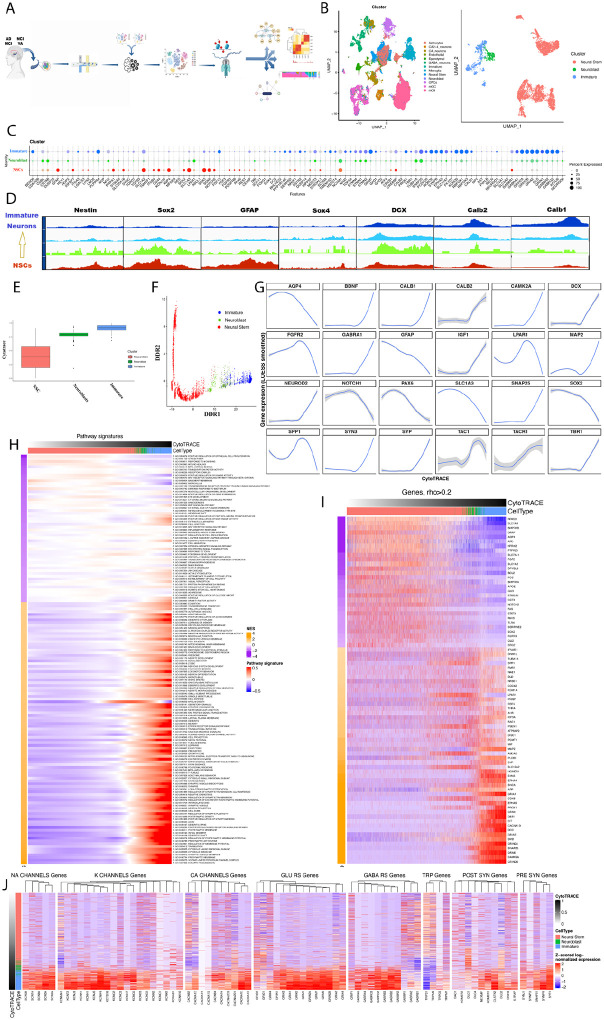
Multiome - based profile of human hippocampal neurogenesis in adulthood. **A**.Schematic illustration of the multiome snRNA-seq/snATAC-seq analysis workflow.**B**. UMAP visualization of 116068 sequenced nuclei clustered using the transfer learning algorithm scANVI cross-referencing annotations from two scRNA-seq datasets (Left panel). A focused UMAP of hippocampal neurogenesis (Right panel). **C.** Dot plot shows selected proxies expressed in neural stem cells, neuroblasts and immature neurons. **D**. Integrative Genomic Viewer (IGV) plots of chromatin accessibility profile of selected proxies on the developmental trajectory of neurogenesis. **E,F**. CytoTrace trajectory (E) and Pseudotime analysis (F) of the neurogenic lineage. **G**. LOESS-smoothed gene expression signatures over CytoTrace trajectory. **H**. Pathway enrichment using GSEA on the GOBP database as a function of CytoTrace in neurogenesis. **I.** Differential gene expression across CytoTrace trajectory in neurogenesis. **J.** Enrichment of transcripts that play a role in excitability, channel activity, and synaptic plasticity in neurogenesis, as a function of CytoTrace.

The transcriptional and epigenetic mechanisms underlying the generation of new neurons from neural stem cells in the subgranular layer of the dentate gyrus (DG) are well established in rodents^11^. Neurogenesis is tightly regulated by the local circuitry, specifically, GABAergic input^12,13^. Parvalbumin-expressing interneurons (PVs) in the DG regulate neurogenesis in an activity- dependent manner^14^. Immature neurons form transient but strong connections with inhibitory neurons in the DG and CA3, which are important for learning^15^. Hippocampal neurogenesis plays critical roles in learning and memory in the rodent brain^1,2^. Immature neurons get recruited into memory circuits and play important roles in memory formation^9,16–18^. Neurogenesis is reduced in the aging rodent, impaired in mouse models of Alzheimer’s disease (AD) and contributes to memory deficits^8–10,19,20^. In contrast, little is known regarding the fate of neurogenesis in the human brain, let alone the mechanisms that regulate it or its function in cognition. The existence of hippocampal neurogenesis in the adult human brain has generated controversy over the past few years^21–26^, primarily attributable to the limitations in the use of species- specific neurogenic proxies, sample processing, cell annotation and computational analysis^27–29^. Other claims suggested that immature neurons were mistaken for inhibitory neurons^23^. Using a machine-learning approach, a recent study identified the existence of immature neurons in the adult human brain^24^ and validated reduced number of immature neurons in Alzheimer’s disease (AD)^25,30^. However, the human DG is thought to be resilient to the development of pathology^31^ and it is not clear what underlies putative shifts in the profile of neurogenesis in aging and AD. Here, we aimed to unravel the molecular and cellular signals that regulate neurogenesis in the adult human brain, and alterations to these signals and the neurogenic niche in aging and AD. To gain an insight into a possible association between neurogenesis and cognitive function, we applied joint single nuclei RNA sequencing (snRNAseq) and single nuclei Assay for Transposase-Accessible Chromatin (snATACseq) on nuclei isolated from the hippocampus of young adults (YA, n=2), aging with no cognitive impairments (NCI, n=4), mild cognitive impairments/early dementia (MCI, n=4) and Alzheimer’s disease dementia (AD, n=4) (Figure 1A, **Supplemental Table 1**).

### Characteristics of neurogenesis in the human brain

In this pipeline, 116,068 nuclei were sequenced. Unsupervised clustering based on snRNAseq revealed 13 cell types in the hippocampus including radial glia/neural stem cells (NSCs), neuroblasts and immature neurons (Figure 1B). To ensure unbiased cell annotation, we utilized the machine learning label transfer algorithm scANVI (https://doi.org/10.15252/msb.20209620) to transfer labels from two scRNAseq datasets, a human developmental forebrain^32^ and an adult human hippocampal dataset^24^. We identified a total of 5,374 radial glia/neural stem cells (NSCs), 416 neuroblasts and 1,179 immature neurons (Figure 1B,C). NSC expression profile included stemness proxies, such as *Slc1A2,3, Notch2, SoxB1 (Sox2,3), SoxD* genes (*Sox5, Sox6), SoxE (Sox9*), radial glial-like proxies (*GFAP, Aqp4*), neural development proxies (*Pax6, Ncam1*) in addition to well-documented adult NSCs’ proxies (*Prrx1, Rest, Lpar1 and Pdgfrb*) (Figure 1C). Neuroblasts had a wide spectrum of proxies, sharing some with NSCs and others with immature neurons. Compared to neuroblasts, immature neurons expressed reduced levels of *Stmn1, Stmn2* and higher levels of *Prox1, Tbr1, Calb1*, *Ncam1, Dcx, Nnat*) and synaptic plasticity and neuronal markers (*Snap25, Synpr, Rbfox1,3, Kcnq5*) (Figure 1C). Further, we examined the pattern of open chromatin events of key neurogenic proxies and observed that stemness signals, such as *Nestin, Sox2 and Gfap* show more open chromatin events in NSCs, while *Dcx, Calb2* and *Calb1* exhibit more open chromatin in immature neurons (Figure 1D). To further phenotype the identified neurogenic population, we performed lineage trajectory analysis and tested if these clusters follow a developmental lineage progression of hippocampal neurogenesis. CytoTRACE analysis revealed linear developmental trajectory where the NSC cluster appeared earlier in development compared to neuroblasts and immature neurons (Figure 1E). Pseudotime analysis validated that these cell types follow a continuous developmental pathway (Figure 1F). Next, we examined the expression pattern of proxies known to transiently peak at different time points in neurogenesis. LOESS-smoothed expression patterns showed that stemness proxies peaked early and were downregulated later in neurogenesis, while differentiation and neuronal maturation proxies were expressed at low levels at an early stage and peaked later in the CytoTRACE trajectory (Figure 1G). Pathway enrichment analysis against CytoTRACE time using GSEA on the NIGO pathway database^33^ revealed that genes and pathways involved in stem cell development were enriched earlier across the trajectory while pathways involved in neuronal maturation, morphology, differentiation, and synaptic plasticity are enriched later across the trajectory (Figure 1H,I, **Supplemental Table 2,3**). In the mouse subgranular zone (SGZ), signals that regulate excitability and ion channel activity were absent early in NSC stage but enriched following fate commitment and neuronal differentiation^34^. Similarly, our analysis revealed that the expression of these transcripts known to convey synaptic function and plasticity, is upregulated during the neuroblast stage (Figure 1J). Together, these observations determine three neurogenic populations, NSCs, neuroblasts and immature neurons among the cellular constituents of the human hippocampus.

### Transcriptomic and Epigenetic modifications drive altered profile of neurogenesis in aging and AD.

**Figure 2:**
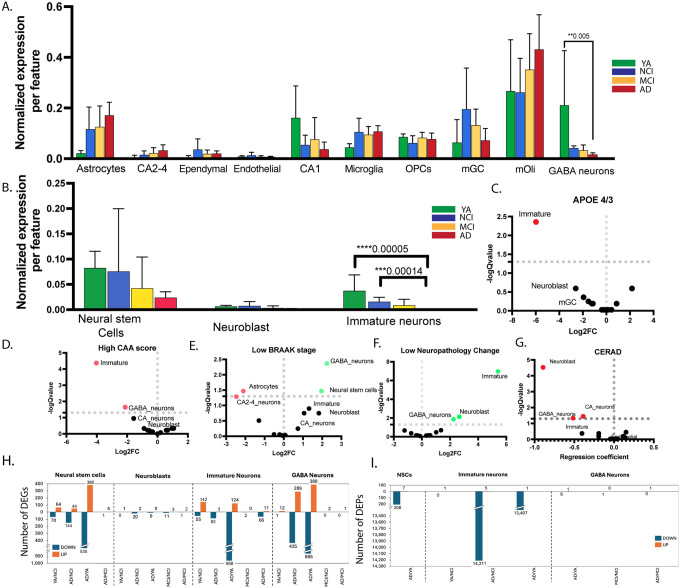
Transcriptomic and Epigenetic modifications drive the profile of neurogenesis as a function of age and Cognitive diagnosis. **A,B**. Normalized expression per feature of the non-neurogenic (A) and neurogenic (B) cells in young adults (YA), No Cognitive Impairments (NCI), Mild Cognitive Impairments (MCI) and Alzheimer’s disease (AD). Values are expressed as a fraction of the total number of cells in the hippocampus. Pairwise comparison between groups. P-values were adjusted using the false discovery rate (FDR) correction (Qvalue), **q < 0.05, *** Q< 0.00014, **** P < 0.00005. **C-G.** Volcano plots of the association of cell abundance with *APOE 4 allele* (C), High CAA score (D), Low BRAAK stage (E) Low neuropathologic change (F) and CERAD score (G). **H,I.** The number of differentially expressed genes (H) or peaks (I) in neurogenesis across diagnoses. Cell types with significant positive (green) or negative (red) associations, q < 0.05.

To examine a putative association between hippocampal cells, age and cognitive function, we asked whether the number or profile of these cells changed with age or cognitive diagnosis. Quantification of cell types revealed that the relative proportion of inhibitory neurons (“GABA_neurons”) and immature neurons were significantly different between diagnoses (q = 0.0258 for each) (Figure 2A,B, **Supplemental Table 4**). Immature neurons (q = 0.000149, p = 1.15E-5), were the only cell type that was significantly reduced in AD compared to NCI (Figure 2A,B). We confirmed the downregulation of immature neurons in AD using a separate donor cohort by immunohistochemistry and unbiased stereology (**Supplemental Figure 1 and Supplemental Table 5**). The number of neuroblasts (q = 0.0812, p = 0.012506) and NSCs (q = 0.2, p = 0.046) in AD compared to NCI were trending lower but did not reach statistically significance. Compared to their numbers in YA, inhibitory neurons (q = 0.005072) and immature neurons (q = 5.21E-05) were significantly reduced in AD. The number of CA1 neurons was trending but not statistically significant (p = 0.045, q= 0.142). Immature neurons were the only cell type that was reduced, trending but not statistically significant, in AD compared to MCI (q = 0.1647, p = 0.012672). The numbers of astrocytes, but not of microglia, were increased in AD, albeit not in a statistically significant manner (Figure 2A,B). Notably, previous attempts have been made to discriminate between NSCs and astrocytes in the rodent and human brains^35–37^. Our snRNAseq-based sequencing, machine-learning analysis and cell annotation based on both developmental and adult human hippocampal dataset revealed distinct clusters of mature astrocytes and NSCs (Figure 1B). The astrocyte cluster showed a trending increase of astrocyte numbers in AD compared to YA (q = 0.09, p = 0.022), while the numbers of NSCs in AD compared to NCI (q = 0.2, p = 0.04) and YA (q = 0.18, p = 0.09) were trending decrease (Figure 2A,B). Three proxy-based immunohistochemistry and unbiased stereology analyses showed that the number of NSCs was comparable in NCI, MCI and AD, while the number of astrocytes increased in AD compared to NCI and positively correlated with high AD pathology (NIA-Reagan criteria, **Supplemental Figure 2**).

To further investigate a possible association between the cell types in the hippocampus, age and diagnosis we examined whether the abundance of the DG cellular constituents correlated with AD pathological hallmarks. We observed that the extent of inhibitory neurons, immature neurons, neuroblasts, neural stem cells (NSCs) and astrocytes was associated with pathological hallmarks. Specifically, the number of immature neurons was greater in *APOE e3; e3* homozygote carriers compared to *APOE e3; e4* (Figure 2C). The number of immature neurons and inhibitory GABAergic neurons inversely correlated with the severity of cerebral amyloid angiopathy (CAA) (Figure 2D). The abundance of NSCs and inhibitory GABAergic neurons positively correlated with low BRAAK stage, while the number of astrocytes negatively correlated with it (Figure 2E). Likewise, the number of neuroblasts and immature neurons correlated with low neuropathology change (Figure 2F). In agreement with that, we observed an inverse correlation between the number of immature neurons and extent of neurofibrillary tangles, and positive correlation with the slope of global cognitive score (**Supplemental Figure 1**). Finally, the number of neuroblasts, inhibitory GABAergic neurons and CA1 neurons correlated with lower cortical neuritic plaque density (CERAD score) (Figure 2G).

To gain insight into the mechanisms of altered cell numbers, we sought to examine the profile of neurogenic cells and inhibitory neurons in YA, NCI, MCI and AD (**Supplemental Table 6,7**). Quantification of differentially expressed genes (DEGs) and peaks revealed that in NSC, a comparable number of DEGs were either up- or downregulated in YA or AD compared to NCI. In immature neurons, the majority of DEG were upregulated in YA compared to NCI. However, in AD, the majority of DEGs expressed in immature neurons were downregulated compared to NCI (Figure 2H). Specifically, In NSC, 64 DEGs were upregulated in YA/NCI while 70 were downregulated. In AD, 44 genes were upregulated while 144 were downregulated in NSC, compared to NCI (Figure 2H). In immature neurons, 197 were DE between YA and NCI, of them, 142 were upregulated in the YC. However, in AD, the majority of DEGs in immature neurons were downregulated. In GABA neurons, the majority of DEGs were downregulated in AD compared to NCI, with 435 genes downregulated and 289 upregulated (Figure 2H). Peak analysis revealed that 208 peaks were downregulated in NSC, but the majority of alterations in open chromatin events took place in immature neurons in AD compared to NCI; 14,211 peaks were downregulated in AD compared to NCI in immature neurons while only 5 were upregulated (Figure 2I). Only a few were significantly altered in inhibitory (Figure 2I). In NSCs we observed 208 peaks downregulated and only 7 upregulated. No significant alterations in open chromatin were observed in neuroblasts among diagnoses. This may be due to the low number of the neuroblasts population. In the case of NSC and inhibitory neurons, this may suggest that most of the alterations we could detect in these cells were of existing transcripts. Together, our data implies that first, a concerted action of transcription and epigenetic mechanisms lead to an altered profile of neurogenesis as a function of age and diagnosis. Second, substantial epigenetic alterations take place in immature neurons compared to NSCs or inhibitory neurons. Third, most transcriptional alterations in neurogenesis takes place in AD.

### Neural stem cell profile is altered in aging

**Figure 3:**
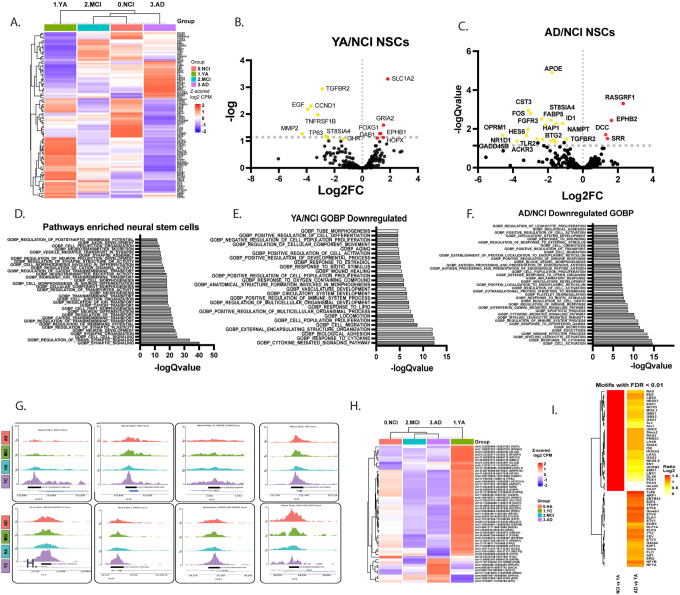
Dynamics of Neural stem cell profile across age and diagnosis. **A**. Heatmap showing pseudo bulk analysis of differentially expressed MANGO genes in neural stem cells across diagnosis (FDR<0.25). **B,C**. Volcano plot of top differentially expressed MANGO genes in YA (B) or AD (C) compared to NCI in neural stem cells (FDR<0.05). **D**. Top significantly enriched GOBP pathway in neural stem cells compared to all other cell types in the hippocampus, (FDR<0.05). **E,F.** Top differentially enriched GOBP pathway in neural stem cells in YA (E) or AD (F) compared to NCI, (FDR<0.05). **G**. IGV tracks of top differentially expressed peaks in neural stem cells across diagnosis (FDR<0.25). **H**. Pseudobulk analysis of differentially expressed MANGO peaks across diagnosis in neural stem cells (FDR<0.25). **I**. Top differentially expressed motifs in neural stem cells in NCI (left) or AD (right) compared to YA FDR<0.01.

To gain an insight into key mechanisms regulating neurogenesis as a function of age and diagnosis, we first examined the profile of NSCs*. Mammalian Adult Neurogenesis Gene Ontology* (MANGO) were significantly enriched in NSC DEGs (q = 2.84X10^−9^). Examination of neurogenesis-related signaling in NSC across our cohort revealed that the transcription profile of YA is distinct of the other 3 diagnostic groups (Figure 3A). The transcription profile of NSCs undergoes modifications in NCI and MCI, leading to an AD profile where most genes showed the opposite expression pattern compared to YA (Figure 3A, **Supplemental Figure 3, Supplemental Table 6–9**). Top DEGs in YA that were downregulated in NCI included genes that play a major role in neuronal function, such as the glutamate transporter Slc1a2, Glutamate Ionotropic Receptor AMPA Type Subunit 2 *Gria2 and Dab1*, critical for brain development and neuronal migration (Figure 3B). Top downregulated DEGs in AD NSCs were linked to self-renewal (*Nampt, Id1*) cell proliferation (e.g., *Cst3, Fgfr3, Fabp5*), and differentiation (*ApoE, Fos, St8sia4*) (Figure 3C). Pathways enriched in the NSC cluster compared to all other cell types in the hippocampus included signal transduction, cell proliferation, nervous system development and cell adhesion (Figure 3D). In agreement with DEGs, top downregulated pathways in NSC in NCI and AD were cellular signaling, proliferation, differentiation, migration, regulation of cell death and inflammatory pathways (Figure 3E,F). Similarly to the DEGs pattern, we observed substantial downregulation of peaks in NCI compared to YA, and this effect was further pronounced in AD compared to NCI (Figure 3G,H). Critical signals, such as *Sox1, Fgf2, Hes5, Apc, Dicer1-as1* and others had reduced open chromatin pattern in their promotors in NCI and showed further closing of chromatin in MCI and AD (Figure 3G,H, **Supplemental Table 10**). Examination of overrepresented motifs in these downregulated peaks inferred regulatory programs that are lost relative to the YA. Notably, many transcription factors were enriched in downregulated peaks in NCI relative to YA, with further downregulation in AD, e.g., *Rax2, En2, Lbx2*. Most of these transcription factors play roles in neurogenesis, brain development (e.g., *Rax, En2, Hesx1, Noto, Gbx1, Arx, Uncx*), growth and differentiation (*lbx2, prrx1, lhx9*) and mitochondrial function (*gabpa*) (Figure 3I). We identified additional motifs, e.g., *Tcfl5, Nrf1*, which represent additional factors whose functionality was lost specifically in AD. Together, these results indicate that substantial regulatory controls in NSCs are lost with aging, with further exacerbation in MCI and AD.

### Alterations in Neuroblast Profile are Pronounced in MCI

**Figure 4:**
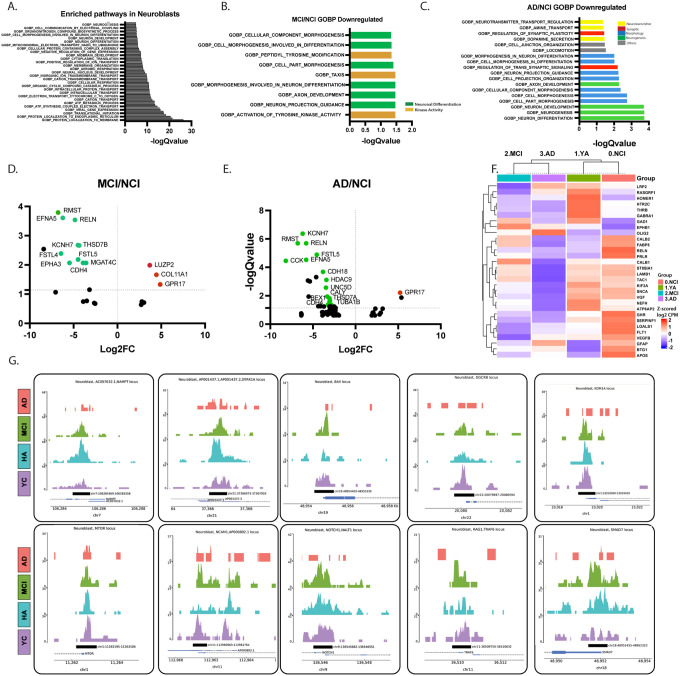
Alterations in Neuroblast Profile are Pronounced in Mild Cognitive Impairments. **A**. Top significantly enriched GOBP pathway in neuroblasts compared to all the other cell types in the hippocampus (FDR<0.05). **B,C**. Top significantly enriched GOBP pathways in neuroblasts in MCI (C) or AD (D) compared to NCI, (FDR<0.05). **D,E**. Volcano plots of top differentially expressed genes in neuroblasts in MCI (D) or AD (E) compared to NCI, (FDR<0.05). **F**. Heatmap of the pseudobulk analysis showing the differentially expressed MANGO genes across diagnosis in neuroblasts (FDR<0.25). **F**. IGV tracks of the top differentially expressed peaks in neuroblasts across diagnosis (FDR<0.25).

In the rodent, neuroblasts are committed to a neuronal fate and give rise to immature neurons^1,38^. However, there is no information on their profile in the human brain.We identified a small population of neuroblasts. Pathway enrichment in neuroblasts revealed pathways of neuronal differentiation, memory, synaptic vesicle exocytosis, and long-term synaptic potentiation were enriched in neuroblasts (Figure 4A). Next, we asked whether their profile changes with cognitive diagnosis. While alterations in neuroblast transcriptomic profile took place between all four diagnostic groups, substantial differences were observed between NCI and MCI (Figure 4B–F, **Supplemental Figure 4, Supplemental Table 6–9**). Top DEGs and pathways downregulated in MCI compared to NCI were associated with neuronal differentiation (Figure 4B,D). Additional pathways that were downregulated in AD compared to NCI were related to synaptic plasticity, neuronal differentiation and neurogenesis (Figure 4C,E,F). Interestingly, we detected only few alterations in neuroblast epigenetic profile. Top differential open chromatin peaks that were upregulated in NCI compared to YA, were downregulated in AD, such as in the *promoters of Nicotinamide Phosphoribosyltransferase (Nampt*), the proliferation and brain development factor *Dual Specificity Tyrosine Phosphorylation Regulated Kinase 1A (Dyrk1A). Neural cell adhesion molecule 1 (Ncam1), Recombination Activating 1 factor (Rag1), Smad Family Member 7 (Smad7), Mtor (Mechanistic Target of Rapamycin Kinase) and Bax* exhibited alterations in open chromatin peaks as a function of age and diagnosis. The microRNA regulator *Dgcr8*, and the *histone deacetylase component Lysine Demethylase 1A (Kdm1a) and Notch1* showed increased closed chromatin in AD (Figure 4G, **Supplemental Table 7**). Taken together, our data suggests that neuroblasts are committed to a neuronal differentiation path, but critical signals and pathways of neurotransmitter regulation, synaptic morphology and neurogenesis were compromised with pathology, with substantial alterations taking place in transcription in MCI and further in AD. This suggests that alterations in the profile of neuroblasts are an early biomarker of cognitive deterioration.

### Shutdown of transcription machineries in Immature neurons in AD`

**Figure 5:**
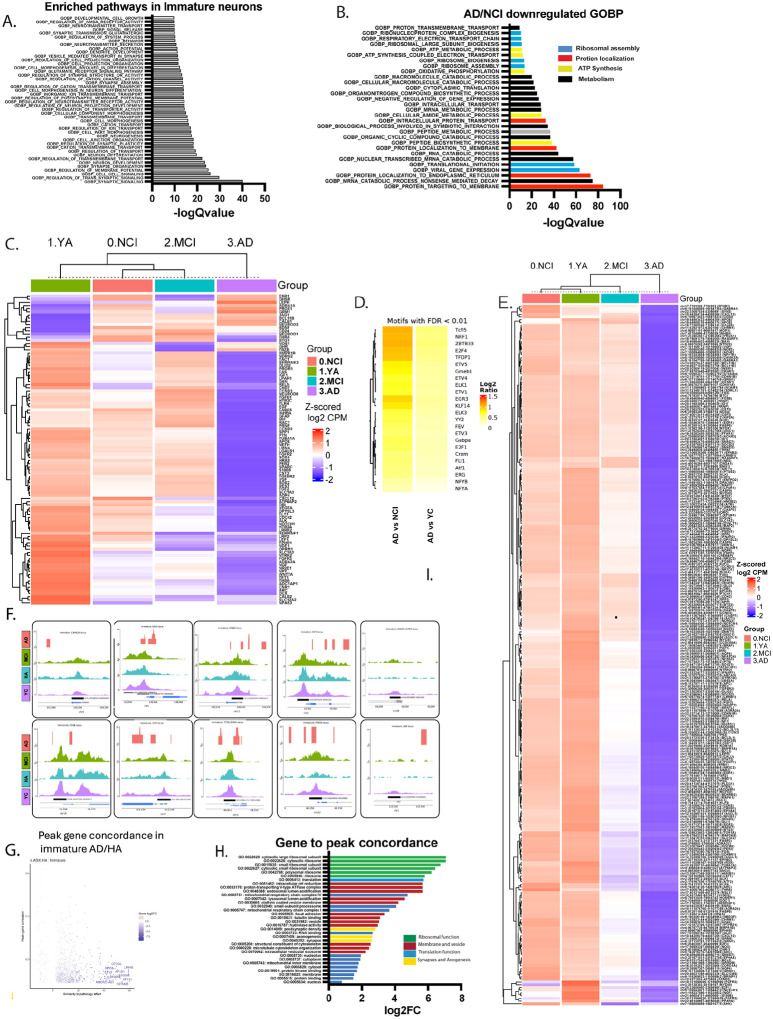
Cessation of transcription machineries in immature neurons in AD. **A**. Divergent Bar plot showing the top significantly enriched GOBP pathways in immature neurons, (FDR <0.05). **B**. Top significantly downregulated GOBP pathways in immature neurons in AD compared to NCI, (FDR <0.05). **C**. Heatmap of pseudo bulk analysis showing differentially expressed MANGO genes in immature neurons across diagnoses (FDR<0.25). **D**. Top differentially expressed motifs in immature neurons in AD compared to NCI or YA (FDR<0.01). **E**. Pseudo bulk analysis of differentially expressed MANGO peaks in immature neurons across diagnoses (FDR<0.25). **F**. IGV tracks of the top differentially expressed peaks in immature neurons across diagnosis (FDR<0.25). **G**. Scatterplot of peak-gene concordance in immature neurons in AD compared to NCI. **H**. Divergent bar plot of pathway enrichment of gene-peak concordance in immature neurons in AD compared to NCI, (FDR<0.05).

In the rodent brain, immature neurons incorporate in the hippocampal circuitry and play a role in hippocampal plasticity. A previous study showed transcriptional dynamics of human immature neurons across the lifespan and showed reduced numbers of immature neurons in AD. However, little is known about their dynamics as a function of cognitive diagnosis. Examination of the profile of immature neurons revealed substantial downregulation of gene expression, including downregulation of most transcripts, peaks and motifs in AD. Specifically, 120 significantly downregulated GOBP pathways in AD compared to NCI (**Supplemental Table 11**, q <0.05). Pathways enriched in immature neurons were neuronal synaptic plasticity, learning and memory and neurogenesis (Figure 5A). DEG and GOBP analyses revealed substantial downregulation of transcripts and their pathways in AD (Figure 5B,C). Top downregulated pathways were mitochondrial and ribosomal (Figure 5B). A comparison of pathways downregulated in AD compared to MCI in immature neurons revealed downregulated calcium regulation and release, serotonin regulation, cognition, brain function and neurogenesis (q <0.05). Likewise, the gene expression profile of immature neurons in the different diagnoses revealed a vastly different gene profile of immature neurons in AD compared to YA, NCI and MCI. Early alterations in gene expression in immature neurons took place between YA and NCI and included major players in neurogenesis, such as *reln, Npy, Calb1,2, Prom1, NeuroD1, Bdnf*. Similarly to enriched pathway, the vast majority of differentially expressed genes were downregulated in immature neurons in AD compared to YA and NCI, such as *Ptn, S100b, Fgfr2, Dcx, Tgfbr2, Reln* (Figure 5C, **Supplemental Figure 5**). Peak and motif profile per diagnosis revealed severely compromised open chromatin events, including of critical neurogenic genes, such as *CamkIIa, Egr1*, *Efnb2, Fosb, Ngf, Fmr1, E2f3, Kif3a, Vgf* in immature neurons in AD (Figure 5D–F). Gene-to-Peak concordance was 9% in immature neurons comparing AD to YA profile, 2% when comparing AD to NCI and 2% in YA compared to NCI. In AD/NCI, most of the concordant peak-gene pairs showed positive correlation and were downregulated in AD (Figure 5G). Gene-peaks of positive concordance play a role in ribosomal structure and function, extracellular vesicular exosome, synaptic density and axogenesis. (Figure 5H). Taken together, our data suggests a considerable cessation of the transcription machineries in immature neurons in AD.

### Profile of inhibitory neurons in relation to age and diagnosis

**Figure 6:**
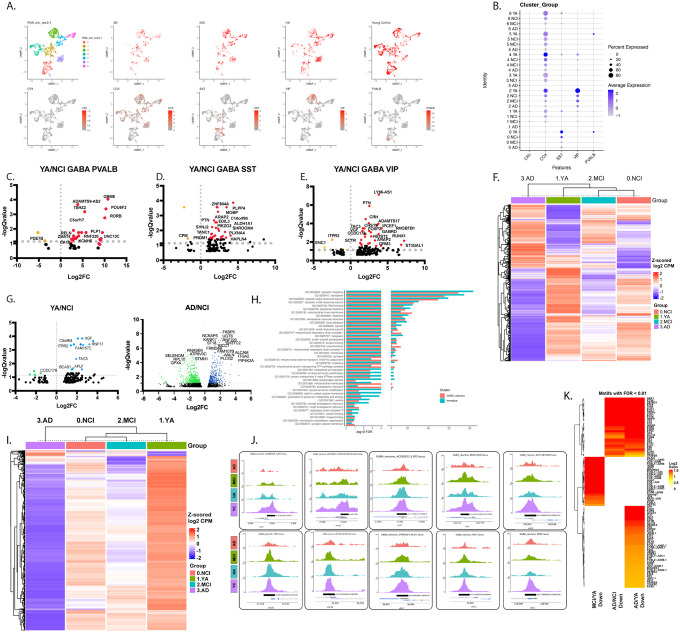
Substantial loss of inhibitory neurons in aging and common pathways with immature neurons. **A**. UMAP plots of total and sub-clusters of inhibitory neurons, *PVALB, SST, CCK, VIP*, and *CR1* across diagnoses. **B**. Dot plot of the distribution of *PVALB+, SST+, CCK+ or VIP+* neurons in subclusters and diagnoses. **C-E**. Volcano plots of top differentially expressed genes in *PVALB+* (C), *SST+*(D), *VIP+* (E) inhibitory neurons in YA or AD compared to NCI (FDR<0.05). **F**. Pseudo bulk analysis of differentially expressed genes in inhibitory neurons across diagnoses (FDR<0.25). **G.** Volcano plots of top differentially expressed genes in all inhibitory neurons in YA or AD compared to NCI (FDR<0.05). **H**. Common significantly downregulated GOBP pathways in inhibitory and immature neurons in AD compared to NCI (FDR<0.25). **I**. Pseudo bulk analysis of differentially expressed peaks in inhibitory neurons across diagnoses (FDR<0.25). **J**. IGV tracks of the top differentially expressed peaks in inhibitory neurons across diagnoses (FDR<0.25). **K**. Differentially expressed motifs as a function of diagnosis in inhibitory neurons (FDR<0.01).

In addition to neurogenesis, our analysis uncovered major alterations in GABAergic neurons in the hippocampal circuitry as a function of age and impairments. Our analysis then identified an acute 4-fold decrease in the number of GABA neurons in NCI, and an additional 2-fold decrease in AD donors (Figure 2A). To gain an insight into the nature of loss of inhibitory neurons, we examined their sub-clusters. In addition to the age- and diagnosis- dependent reduction in total number of inhibitory neurons, we observed that parvalbumin+ (PValb+) neurons were completely lost in NCI (Figure 6A,B), and Vasoactive Intestinal Peptide (VIP)+ and somatostatin (SST)+ were robustly downregulated in NCI compared to YA (Figure 6A,B). Examination of DEGs in subclusters and in PValb+, VIP+ and SST+ neurons in NCI compared to YA revealed downregulation of several signals that play a role in synaptic plasticity. For example, in PValb+ neurons, signals such as metabotropic receptor *Grm8*, the voltage gated potassium channel *Kcnh5, Unc13c* predicted to play a role in calmodulin and syntaxin binding activity and the extracellular matrix signal reelin were downregulated (Figure 6C); in SST+ neurons, we observed downregulation of signals, such as *Syt10*, *Sema3C, Kcnh1 and Plxna4* (Figure 6D); in VIP+, downregulation of *Gabrd, Sulf1, Epb41, Grik3* (Figure 6E). Examination of the overall profile of inhibitory neurons revealed alterations in transcription as a function of age and diagnosis with marked alterations in AD. DEGs and Pathway enrichment analysis in AD compared to NCI revealed pathways essential for myelin regulation, transmembrane transport, ATP and energy production, mitochondrial function, and translation (Figure 6F–H, **Supplemental Figure 6, Supplemental Table 12**). Interestingly, there was a large overlap of DEGs in common between Immature and GABAergic neurons, leading to common pathways. Specifically, there were 261 genes that were differentially expressed in AD/NCI in both immature and GABAergic neurons; 245 of them were changing concordantly and 236 were concordantly downregulated. A total of 439 genes were downregulated in Immature neurons, 1446 down in GABA neurons, 236 downregulated in both, out of 32,000 total genes tested in the genome (Odds ratio 29.1, Fisher’s Exact test p-value 8.2e-200). Further, 40 of the downregulated pathways in immature and GABAergic neurons were in common (Odds ratio 424, Fisher’s Exact test p-value 1.7e-64). This may suggest a similar system-level effect between immature and inhibitory neurons in the transition from NCI to AD (Figure 6H, **Supplemental Table 13**). Interestingly, the majority of peaks in GABA neurons were substantially downregulated in AD compared to other diagnoses, suggesting marked closure of chromatin in these neurons (Figure 6I–K, **Supplemental Table 14**). Notably, in contrast to DEGs, the number of DE peaks was substantially different between immature and inhibitory neurons. While in the former we observed a large number of differential peaks (41,341 in AD/NCI, 94 up and 41,247 down; 67,532 total in AD/YA, 67 of them were up and 67,465 down), in GABA neurons the effect was smaller. There were 1659 DE peaks in AD/YC (39 up, 1620 down) and only 87 in AD/HA (8 up, 79 down) (Figure 6I,J). Interestingly, of DE peaks in AD/YA, 1463 of the downregulated pathways were in common for immature and inhibitory neurons (Fisher’s Exact test *P* = 1.4e-232, **Supplemental Figure 7 and Supplemental Table 15**). Examination of motifs in inhibitory neurons showed 68 strongly enriched (log2 odds ratio >1, FDR<0.01) motifs for downregulated peaks in AD/YC in these neurons, with earliest alterations in MCI compared to YA (Figure 6K). Together, our data suggests an age-dependent loss of inhibitory neurons in the hippocampus, characterized, in part, by loss of synaptic plasticity signalling, a set of processes led by transcription, accompanied by some chromatic closure in MCI and AD.

### Neurogenic cell type communication with the cellular environment

**Figure 7:**
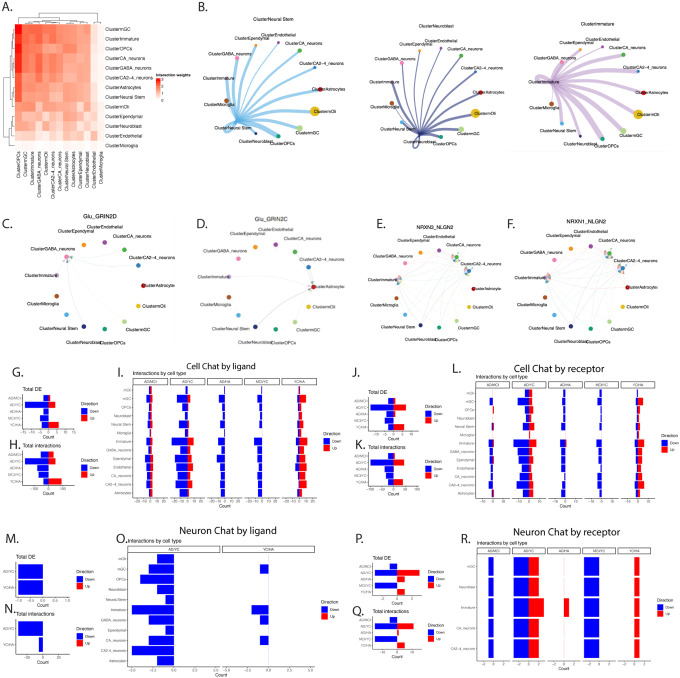
Cell communication in the neurogenic niche of the hippocampus. **A**. CellChat cluster interaction heatmap shows the strength of cluster-to-cluster interactions. **B**. Network plot of intercellular communication between neural stem cells (left panel), Neuroblast (middle panel) and immature neurons (right panel) with other cell types in the hippocampus. String width represents the strength of the communication. **C-F**. Representative network plots of interactions of *Glutamate_GRIN2D* (C), *Glutamate GRIN2C* (D) and *NRXN3_NLGLN2*(E) *NRXN1_NLGN2* (F). **G-L**. Cell Chat analysis by ligand (G-I) or receptor (J-L) of the total number of differentially expressed ligands (G,J), total interactions (H,K) in immature neurons or in each cell type (I,L) for each intra-diagnosis comparison. Up or down-regulated changes are delineated by the color and the direction of the bar FDR<0.25). **M-R**. Neuron Chat analysis by ligand (M-O) or receptor (P-R) of the total number of differentially expressed receptors (M,P), total interactions (N,Q) in immature neurons or in each cell type (O,R) for each intra-diagnosis comparison. Up or down-regulated changes are delineated by the color and the direction of the bar FDR<0.25).

To start to address a putative interaction of neurogenesis with their cellular niche in the human brain, we attempted to predict cell interaction by examining ligand-receptor co-expression using CellChat^39^ and NeuronChat^40^. The strongest interaction of neurogenesis was with oligodendrocyte precursor cells (OPCs) and inhibitory neurons (Figure 7A,B). Interaction analysis showed that NSCs, neuroblasts and immature neurons are predicted to communicate with all neighboring cells in the DG via cell adhesion molecules, ligand-receptor interaction, neurotransmitter secretion, synaptic adhesion molecules and the secretion of neuropeptides (**Supplemental Tables 16,17**). For example, immature neurons and neuroblasts secrete glutamate, that binds GRIN2D receptors expressed in inhibitory neurons and astrocytes (Figure 7C,D). Similarly, several cell types, including neuroblasts and inhibitory neurons secrete *NRXN1,3* which binds *NLGN2* receptors in immature and CA neurons (Figure 7E,F). These pathways play critical roles in synaptic plasticity and are implicated in cognitive deficits and AD dementia^41–44^. These analyses further inferred ligand-receptor pathways that may play a role in a decline in cell-cell interaction as a function of pathology in immature neurons, as well as ligands and receptors that may be exclusively expressed in immature neurons (**Supplemental Figure 8, Supplemental Tables 16,17**). Specifically, cross reference of DEGs to the interactions from CellChat and NeuronChat revealed that there were no significant alterations in cell-cell interactions because of altered ligands secreted by immature neurons in NCI compared to YA(**Supplemental Tables 16,17**). However, several ligand-receptor pathways led a decline in cell-cell interaction as a function of pathology in immature neurons. Particularly, *Efna5,Ptn, Lama2, Sema5a,6D* ligands secreted by immature neurons. These ligands bind receptors expressed by several cell types in the hippocampus (**Supplemental Figure 8A-F**). This analysis further revealed ligands that were exclusively expressed in immature neurons. Unique ligands included *Pdgfd, Postn, Efnb3, L1cam*, *Lrrc4, Bmp7, Cntn2, Ncam1, Nectin3* (**Supplemental Figure 8G-S**). Interestingly, we found high gene-peak concordance for *Ephb6, 1, Epha4, Efnb2,3, Oprm1, Oprd1 and Pdyn*, validating a concordance of open chromatin regions and transcription factors that could regulate expression of these ligands and receptors. Total cell-cell interaction in relation to diagnosis revealed that in most cell types in the hippocampus, the number of ligand and receptor interactions in YA was greater compared to NCI (Figure 7G,J). In contrast, a marked reduction in ligand - receptor interactions takes place in MCI compared to YA and in AD compared to NCI (Figure 7G,J). The number of differentially expressed ligands or receptors in immature neurons as well as total interactions in YC was largely upregulated compared to NCI (Figure 7H,I,K,L). In contrast, interactions were largely downregulated in AD compared to the other diagnoses (Figure 7H,I,K,L). Neuron-specific ligand-receptor analysis revealed that most interactions were downregulated in immature neurons in MCI compared to YA, suggesting that reduced interaction precedes the onset of AD. Interestingly, the total number of interactions to or from each individual cell type by neuronal ligands was lower in selected cell types, i.e., mGC, immature neurons, GABA neurons and CA neurons, in YA compared to NCI (Figure 7M). Interactions were further substantially reduced in all cell types in AD compared to YA (Figure 7M–O). The number of interactions to or from each individual cell type by neuronal receptors was largely reduced in MCI/YA and AD/MCI (Figure 7P,R).

## Discussion

This study unravels a comprehensive transcriptomic and epigenetic signaling network of neurogenesis and the cellular constituents of the hippocampus as a function of age and cognitive diagnosis. The analyses performed here revealed several novel observations. First, we elucidated the epigenetics and transcriptomics profiles of hippocampal neurogenesis. We observed that neurogenesis is differentially governed by a concerted action of both regulatory mechanisms. Alterations in open chromatin where particularly pronounced in immature neurons as a function of diagnosis. Second, we identified three neurogenic populations, NSCs, neuroblasts and immature neurons. Our computational analysis and cell annotation approaches identified mature astrocytes and NSCs as distinct cluster populations. The number of neuroblasts was particularly low compared to the number of immature neurons and NSCs, which may suggest that cells stay as neuroblasts for a short period following which they differentiate into immature neurons. We show that the earliest robust alterations in the profile of neurogenesis took place in NSCs and were age - dependent. Notably, we observed that NSCs exhibit inflammatory characteristics. Inflammatory profile was modulated with aging. The molecular profile of neuroblasts was substantially altered in MCI followed by AD. Together with the observation that the number of neuroblasts inversely correlated neuritic plaque density and with cortical neuritic plaque pathology (CERAD score) suggests that alterations in these cells may represent early changes in AD neurodegeneration. This observation agrees with our previous report^30^. Third, the profile of immature neurons was substantially altered in AD compared to the rest of the diagnostic groups tested here. A distinct profile characterized by a substantial downregulation of most transcripts, peaks and motifs was observed in these cells in AD, as detected by both transcription and epigenetic analyses. Fourth, immature neurons and inhibitory GABAergic neurons are the main cell types whose their abundance significantly changed with age and diagnosis and their numbers inversely correlate with levels of pathology and APOE risk factor alleles, but the profile of all cell types in the hippocampus underwent age- and diagnosis-dependent alterations. Fifth, similar to the rodent, we observed that immature and inhibitory neurons are functionally connected in the hippocampus^4,5^. We show that immature neurons and inhibitory GABAergic neurons have distinct regulatory networks, however, there are clear interactions of these cell types across common pathways. Particularly intriguing were common GOBP pathways and motifs that were downregulated in AD compared to NCI. This provides support that hyperexcitability, described in mouse models of AD, takes place in the human AD brain, and the loss of immature and inhibitory neurons is a major contributor to this phenomenon. Sixth, PV+ inhibitory neurons appeared most vulnerable to aging, and were absent in the NCI, MCI and AD hippocampus. Our observation that inhibitory neurons. Recent studies mapping the cellular landscape of the prefrontal cortex in AD reported loss of SST inhibitory neuron subtypes in AD^45,46^, which we show to be the case in hippocampus even in normal aging. Lastly, we show that NSCs, neuroblasts and immature neurons communicate with cellular counterparts in the hippocampus and that these interactions were severely compromised in MCI and AD, some of which may be attributed to the reduction in ligands and receptors exclusively expressed in immature neurons. Our study provides a comprehensive profile of the human hippocampal cellular residents and insight into their putative functions across age and dementia.

## Materials and Methods

### Donor cohorts and tissues

Where applicable, all human tissue studies were approved by the institutional review boards with oversight over the specific cohort studies and all tissues were obtained with informed consent. Human brain tissue samples for molecular omics/sequencing studies were provided by the University of Washington (UW) BioRepository and Integrated Neuropathology (BRaIN) laboratory, which supports a number of cohort studies that were represented in the cohort for this study including the UW Alzheimer’s Disease Research Center (ADRC) clinical core, the Kaiser Permanente Washington Health Research Institute Adult Changes in Thought (ACT) study, the Seattle Longitudinal Study (SLS), and the Pacific Northwest Brain Donor Network (PNBDN). Tissues were derived through a rapid tissue collection process, performed when postmortem interval < 12 hours, and which includes rapid procurement of the donor brain, coronal slicing and rapid sampling and freezing (either flash freezing in liquid nitrogen or in supercooled dry-ice/isopentane slurry), fixation of brain followed by routine sampling and diagnostic neuropathological analysis according to NIA-AA guidelines for the pathological assessment of AD and related dementias^47,48^. This study was designed with four groups, including young age/healthy donors (YA), healthy aged donors with no or low AD neuropathologic change and normal cognition (NCI), donors transitioning from healthy aging to AD dementia generally with mild cognitive impairment or early dementia (MCI) regardless of pathology (none to high), and donors with AD neuropathology and established consensus diagnoses of dementia (described in **Supplemental Table 1**). Donor tissues for IHC and stereological validation studies were provided by the Rush University School of Medicine Alzheimer’s Disease Research Center Memory and Aging Project, where donors underwent similar neuropathological protocols as those at UW and where the formal-fixed, paraffin-embedded tissue blocks/slides were provided from hippocampus/medial temporal lobe for this study (described in **Supplemental Table 5**, see more details in **Supplemental Methods**).

### Fresh Frozen samples preparation and nuclei isolation

The dentate gyrus was isolated from Fresh Frozen blocks of 14 participants representing 4 diagnostic groups (YA, NCI, MCI and AD). Tissue was then immediately moved to homogenization; For each sample, a separate homogenizer and douncing pestles (loose and tight) were used. Each sample was homogenized in 1ml pre-chilled lysis buffer (0.1% NP-40 Alternative (or NP-40), 10mM Tris, 146mM NaCl, 1mM CaCl2, 21mM MgCl2, 40U/mL of RNAse inhibitor) by mechanical douncing for 20 times with the loose pestle followed by 20 times with the tight pestle to obtain a single cell suspension. Cell suspensions were then incubated on ice for 5 minutes. The homogenate was then filtered through 70 μm filters (Milentyi Biotec 130–041-407) and 40 μm filters (Milentyi Biotec 130–041-406), moved to 1.5 ml tubes and centrifuged at 4C for 5 minutes at 500 RCF. Supernatants were removed and the nuclei pellet was washed 3 times with wash buffer (10mM Tris, 146mM NaCl, 1mM CaCl2, 21mM MgCl2), 0.01% BSA, 40U/mL of RNAse inhibitor). After the last wash, supernatants were removed, and the nuclei pellet was resuspended in resuspension buffer and mixed with 900 μl of Sucrose Cushion buffer. To remove the additional debris, resuspended nuclei were then loaded above a 3 Sucrose Cushion gradient (2.7 ml Nuclei PURE 2M Sucrose Cushion Solution with 300 μl Nuclei PURE Sucrose Cushion Buffer) and the sucrose gradient containing the nuclei was Centrifuged at 13,000 rcf for 45 min at 4°C. The supernatant was then carefully removed and the samples were immediately processed following the Single Cell protocol from 10x Genomics.

### The 10X Genomics multiome library preparation and sequencing

The 10X Genomics Multiome library preparation and sequencing was done at Northwestern University NUseq facility core with the support of NIH Grant (1S10OD025120). Nuclei number was analyzed using Nexcelom Cellometer Auto2000 with AOPI fluorescent staining method. The nuclei were first undergone transposition with ATAC enzyme for one hour at 37°C. we loaded 16,000 transposed nuclei into the Chromium Controller (10X Genomics, PN-120223) on a Chromium Next GEM Chip J (10X Genomics, PN-1000230), and according to the manufacturer’s protocol, single cell gel beads were generated in the emulsion (GEM). Barcoded DNA and cDNA were PCR amplified and subjected to library construction. The single nuclei ATAC-seq library was generated using the Chromium Next GEM Single Cell Multiome ATAC + Gene expression kit (10X Genomics, PN-1000281) and single Index Kit N Set A (10X Genomics, PN-1000212) according to the manufacturer’s manual. In addition, the amplified cDNA was used for the gene expression library using dual Index Kit TT Set A (10X Genomics, PN-1000215). Quality control for the constructed library was performed by Agilent Bioanalyzer High Sensitivity DNA kit (Agilent Technologies, 5067–4626) and Qubit DNA HS assay kit for qualitative and quantitative analysis, respectively. For the snATAC-seq library, the multiplexed libraries were pooled and sequenced on Illumina Novaseq sequencer with 100 cycles kits using the following read length: 50 bp Read1 and 49 bp Read2. For the snRNA-seq library, the libraries were sequenced on Illumina Novaseq sequencer with 100 cycles kits using the following read length: 28 bp Read 1 for cell barcode and UMI and 90 bp Read 2 for transcript expression. The targeted sequencing depth for snATAC-seq and snRNA-seq is 25,000 and 20,000 reads per cell, respectively.

### 10X single-cell multi-ome

Raw reads were demultiplexed and single-nucleus gene expression and peak enrichment were quantified simultaneously using CellRanger-arc count (10X Genomics). The quality of the run was assessed through (1) demultiplexing metrics, including the number of cells captured and the percent of reads with valid barcodes; (2) gene expression metrics, including the percent of mappable reads to the genome and transcriptome, and the median UMI counts and median genes expressed per cell; and (3) open chromatin metrics, including the percent of mappable reads to the genome, within peaks, and to promoter sequences, and the median counts and total peaks observed per cell. Following the quantification and peak calling analysis for each individual sample, all captures were aggregated to obtain a unified feature set for downstream analysis using CellRanger-arc aggr (10X Genomics).

### Single-cell analysis

All samples were analyzed together. Single cells were filtered to ensure data used in the downstream analysis is high quality: cells with >10% mitochondrial expression, indicative of dead or dying cells; cells with low numbers of genes expressed (<1000 genes) or low total UMI RNA counts (<2000 UMI counts); and cells with low peak numbers (<200 peaks) or total ATAC counts (<500 counts) were removed. Clustering was performed on the RNA-seq data, anticipating that gene expression would have a higher dynamic range than open chromatin, using the Seurat package in R^49^. Gene expression was normalized using NormalizeData, and the top 6000 variable genes were identified using FindVariableFeatures, both with default parameters. The top variable genes were z-scored using ScaleData and principal components were computed using RunPCA for the top 200 PCs. Statistical significance of each PC was computed using JackStraw and heatmaps of the top cells and genes per PC were plotted using DimHeatmap; after reviewing both results the top 125 PCs were selected as features for clustering analysis. Clustering analysis was performed with the Louvain algorithm as implemented in Seurat at resolutions 0.25, 0.5, 1, and 2. After reviewing the expression of known marker genes, we based our downstream analysis on the clustering results at resolution 1.

### Cell type determination

In order to ensure consistency of cell annotation, we used a transfer learning approach based on scVI (10.1038/s41587-021-01206-w) and scANVI (doi.org/10.15252/msb.20209620) in which reference dataset annotations are transferred to annotate subpopulations in a new dataset. As one of our key goals was the identification and identification of immature or developing cell populations in the adult human brain, we used one human developmental forebrain scRNA-seq dataset (10.1038/s41586-018-0414-6) as well as one adult human hippocampal scRNA-seq dataset (10.1038/s41586-022-04912-w) as the reference datasets for the annotation transfer to our human adult hippocampal scRNA-seq dataset. The combination of these two datasets allowed us to train a scVI (v1.0.3) model (10.1038/s41587-021-01206-w). The latent representation was modeled using the top 5000 most variable genes in the combined dataset and was corrected for batch effect based on the data source. We then used scANVI (v1.0.3) (doi.org/10.15252/msb.20209620) (n_samples_per_label=500) to transfer the most likely label from the reference datasets to the unannotated cells in our dataset.

### Cell type abundance and statistical analysis

We counted the total cells per cell type per sample and computed association statistics between these cells counts and subject diagnosis and other AD-related traits using edgeR^50^ without the TMM normalization. Comparisons between groups, e.g., cell abundance in AD vs HA subjects, were computed using the exactTest function^50^. Association of cell type abundance with continuous variables, such as cognitive scores, were computed using generalized linear models (GLMs).P-values were adjusted for multiple testing using the false discovery rate correction.

### Differential gene expression between clusters

Differentially expressed genes for each neurogenic cell type were obtained using the FindAllMarkers function in Seurat^49^ with the Wilcox test, comparing each cell type to all other cells. This analysis was performed between NSCs, neuroblasts, and immature neurons only. Significantly differentially expressed genes were determined based on adjusted q < 0.05.

### Differential gene and peak expression by diagnosis

Differential gene and peak statistics between diagnosis groups were computed using a pseudo-bulk approach. Counts for gene expression or open chromatin were summed for each sample across all cells within a given cluster. Low-expressed genes or peaks – expressed in fewer than 25% of samples or with fewer than 50 total counts across all samples – were removed. Differential expression and open chromatin statistics for each cluster were computed using edgeR using the exactTest^50^ to perform pairwise between groups. P-values were adjusted using the false discovery rate (FDR) correction. Differentially expressed genes (DEGs) were determined based on FDR<0.05.

### Motif analysis of DE peaks

Motif enrichment analysis will allow us to infer specific transcription factors that are driving developmental changes between clusters. First, we searched for instances of known transcription factor motifs in all peak sequences from the JASPAR database^51^ using FIMO^52^. Then we computed motif enrichment statistics for each set of DE peaks by comparing the fraction of motif-containing peaks within or not within the DE peaks using Fisher’s Exact Test. We repeated this test for all motifs, correcting for multiple testing using the FDR correction of Benjamini and Hochberg^53^.

### Single-cell trajectory analysis

Cell types related to neurogenesis were isolated from the overall data set, and their relative progress along developmental stages was inferred using CytoTRACE^54^, which uses a robust gene expression diversity statistic to assess cellular differentiation. We paired the time point inference from CytoTRACE with a pseudotime analysis in Monocle2^55,56^ to infer the different paths through the cell populations. We assessed both genes and open chromatin peaks whose expression changes systematically over developmental stages by correlating expression with CytoTRACE time using a Spearman correlation.

### Topic modeling for the neurogenic population

Latent Dirichlet allocation topic modeling was used to model the diverse cell state within the DG neurogenic population using the fastTopics^57,58^ package in R. Gene association for each topic was computed using a specificity log-ratio, defined as the log2 ratio of the highest to the second-highest topic contribution per gene. Genes with specificity log-ratio bigger than 4 were considered the top genes for each topic, and used for pathway analysis.

### Pathway analysis of DEGs

Pathway enrichment of was interrogated against the Neuroimmune Gene Ontology (NIGO) Biological Process (BP) pathway database. Pathway enrichment for differentially expressed genes, or top genes per topic, was performed with Fisher’s Exact test in R. Pathway enrichment for genes associated with CytoTRACE time was performed with GSEA^59^ with 1000 permutations, using the Spearman correlation coefficient as a ranking statistic. P-values were adjusted for multiple testing using the false discovery rate correction.

### Cell-cell interaction

Interactions between cell types was inferred using both CellChat^39^ and NeuronChat^40^ packages in R, in both cases using default parameters. Ligands and receptors were cross-referenced with the differentially expressed gene statistics from the pseudobulk analysis to determine cell-to-cell interactions that may altered by AD pathology.

## Data Availability

Raw and processed data sets associated with this study are available on Gene Expression Omnibus (GEO) at GSE.
